# Optimization of Magnetoimpedance Effect and Magnetic Properties of Fe-Rich Glass-Coated Microwires by Annealing

**DOI:** 10.3390/s23177481

**Published:** 2023-08-28

**Authors:** Alvaro González, Alfonso García-Gomez, Valentina Zhukova, Paula Corte-Leon, Mihail Ipatov, Juan Maria Blanco, Julian Gonzalez, Arcady Zhukov

**Affiliations:** 1 Department of Polymers and Advanced Materials: Physics, Chemistry and Technology, Faculty of Chemistry, University of the Basque Country (UPV/EHU), 20018 San Sebastian, Spain; alvaro.gonzalezv@ehu.eus (A.G.); alfonso.garciag@ehu.eus (A.G.-G.); valentina.zhukova@ehu.es (V.Z.); paula.corte@ehu.eus (P.C.-L.); mihail.ipatov@ehu.es (M.I.); julianmaria.gonzalez@ehu.eus (J.G.); 2 Departamento de Física Aplicada, Escuela de Ingeniería de Gipuzkoa, University of the Basque Country (UPV/EHU), 20018 San Sebastian, Spain; 3 EHU Quantum Center, University of the Basque Country (UPV/EHU), 20018 San Sebastian, Spain; 4 IKERBASQUE, Basque Foundation for Science, 48011 Bilbao, Spain

**Keywords:** magnetic microwires, annealing, hysteresis loop, giant magnetoimpedance, domain wall propagation, magnetic anisotropy

## Abstract

As-prepared Fe-rich microwires with perfectly rectangular hysteresis loops present magnetization reversal through fast domain wall propagation, while the giant magnetoimpedance (GMI) effect in Fe-rich microwires is rather low. However, the lower cost of Fe-rich microwires makes them attractive for magnetic sensors applications. We studied the effect of conventional (furnace) annealing and Joule heating on magnetic-propertied domain wall (DW) dynamics and the GMI effect in two Fe microwires with different geometries. We observed that magnetic softness, GMI effect and domain wall (DW) dynamics can be substantially improved by appropriate annealing. Observed experimental results are discussed considering the counterbalance between the internal stresses relaxation and induced magnetic anisotropy associated with the presence of an Oersted magnetic field during Joule heating.

## 1. Introduction

Magnetic wires with amorphous and nanocrystalline structures have attracted considerable attention due to an unusual combination of magnetic properties, such as magnetic bistability, fast domain wall (DW) propagation and the giant magnetoimpedance (GMI) effect, suitable for numerous applications in magnetic and/or magnetoelastic sensors and devices [1,2,3,4,5,6,7,8]. In addition to excellent soft magnetic properties, amorphous wires also present enhanced mechanical properties, which make them even more attractive for sensor applications [9,10,11]. Generally, amorphous materials also present better corrosion properties [12]. The latter can be improved even more by the insulating, flexible glass coating provided by the Taylor–Ulitovsky technique, allowing preparation of magnetic microwires with an amorphous structure covered with insulating, flexible and biocompatible glass coating [13,14,15]. Such glass-coated microwires with a unique combination of physical properties become therefore suitable for a number of applications, including biomedicine [16,17], electronic surveillance [6,7] or smart composites [18,19,20,21].

Initially, it was thought that diameters of the metallic nucleus of such glass-coated microwires were limited to 5–30 µm; however, preparation of glass-coated microwires from 0.2 to 100 µm has recently been achieved and reported [22,23].

The aforementioned GMI effect is one of the most promising phenomena for development of high-performance magnetic sensors and magnetometers with magnetic field sensitivity up to pT level [2,3,4]. The nature of the GMI effect is commonly attributed to the dependence of skin depth *δ* of a magnetic conductor on applied magnetic fields [24,25,26]. Consequently, substantial GMI effect can be observed in magnetically soft materials. Therefore, the highest GMI effect is reported in amorphous and/or nanocrystalline materials [2,4,14,24,25].

The GMI effect is commonly expressed in terms of the GMI ratio ∆*Z*/*Z*, defined as [14,24,25]:

∆*Z*/*Z* = [*Z* (*H*) − *Z* (*H_max_*)]/*Z* (*H_max_*) × 100 (1)

where: *H*—applied magnetic field, and *H_max_*—maximum applied magnetic field.

The magnetic softness of amorphous materials is principally restricted by the magnetoelastic anisotropy [27,28]. Accordingly, a common method to improve the magnetic softness of amorphous materials is using chemical compositions with vanishing magnetostriction coefficient *λ_s_* [14]. Basically, nearly-zero *λ_s_* values have been reported for Co-rich amorphous materials in (Co_1−*x*_Fe*_x_*)_80_(SiBP)_20_ alloys for 0.03 ≤ *x* ≤ 0.08 [14,28,29,30]. However, Co is one of the critical elements [31]. Therefore, the search for alternative methods for magnetic softness optimization of Fe-rich microwires with amorphous structure is relevant from the viewpoint of technological applications [14,32,33].

Generally, perfectly rectangular hysteresis loops with relatively high coercivity (50–500 A/M) and low magnetic permeability are reported for Fe-rich amorphous microwires with a positive *λ_s_* [14,32,33,34,35,36]. The magnetization reversal of as-prepared amorphous Fe-rich wires runs through a large and single Barkhausen jump [36,37,38,39]. The main interest in such Fe-rich microwires is related to ultrafast magnetization switching upon application of a reversal magnetic field by a single domain wall (DW) propagation with a velocity *v* of up to several km/s [36,37,38,39,40].

However, recently, it has been reported that annealing of Fe-rich microwires in the presence of applied stress (stress annealing) allows substantial improvement of magnetic softness and GMI ratio due to induced magnetic anisotropy [32,33].

Previously, it was considered that the amorphous structure of glass-coated microwires could only be obtained for metallic nucleus diameters *d* below 30 µm [10,14]. However, we recently reported the preparation of completely amorphous glass-coated microwires from Fe_72_B_13_Si_11_Nb_3_Ni_1_ alloy with *d* ≈ 100 µm [14,23]. Such “thick” Fe_72_B_13_Si_11_Nb_3_Ni_1_ microwire presents a good combination of magnetic properties, such as considerable GMI effect and fast DW dynamics, even in an as-prepared state [23]. On the other hand, magnetic softness and GMI effect, as well as DW dynamics, are substantially affected by the postprocessing, such as furnace annealing and Joule heating [34,35,36].

As mentioned above, the magnetoelastic anisotropy is the main source of magnetic anisotropy in amorphous materials [27], and the *λ_s_* sign and value are the principal factors that affect the magnetic softness of amorphous microwires [14,27]. The *λ_s_* values to a great extent are determined by the content of ferromagnetic elements, such as Co, Fe and Ni. Thus, in Fe-rich microwires, positive *λ_s_* values of the order *λ_s_*~40 × 10^−6^ are reported, while, in Co-rich microwires, *λ_s_* is negative (*λ_s_*~−5 × 10^−6^) [28,29,30].

In our previous publications we extensively studied the magnetic properties of Fe_75_B_9_Si_12_C_4_ microwires with *λ_s_* ~ 40 × 10^−6^ [32,33]. On the other hand, recently, we prepared thicker (up to 100 µm) Fe_-_B_-_Si microwires doped with a small amount of Nb and Ni, i.e., with the same *λ_s_* ~ 40 × 10^−6^ [14,23]. It seems that doping with Ni and Nb not only allows large-diameter microwires to be obtained, but also improves their mechanical properties [41,42].

Therefore, the purpose of this paper is to present a comparative study of the effects of annealing on the GMI effect and DW dynamics of conventional Fe-rich microwires and thicker Fe_72_B_13_Si_11_Nb_3_Ni_1_ microwires.

## 2. Materials and Methods

We studied two Fe-rich glass-coated microwires prepared using the Taylor–Ulitovsky technique, described in detail elsewhere [13,14]. This technique involves the rapid quenching of previously molten metallic alloys surrounded by an insulating glass coating. Briefly, a metallic ingot is made molten by high-frequency inductor inside a glass (Duran) tube. Then, a glass capillary is formed and filled with molten metallic alloy. The composite microwire is then pulled out and attached to a rotating receiving spool. Rapid quenching is achieved by a flow of coolant during drawing. Variation in parameters such as wire drawing speed, alloy temperature, glass tube feed rate and spool rotation allows control of the metallic nucleus diameter *d* and total diameter *D*, as detailed in references [13,14,15].

As discussed elsewhere, the difference in the thermal expansion coefficients of the glass coating and metallic alloys is the main source of the internal stresses [13,14,15].

The chemical composition d and D values of the studied samples are provided in Table 1.

It is worth noting that both studied samples had rather similar *ρ*-values. As demonstrated elsewhere [13,14,15], the internal stresses σ value is mainly determined by the difference in the thermal expansion coefficients of the metallic alloys and glass-coated microwires. The main parameter affecting the σ value is the *ρ*-value. However, the metallic nucleus diameter *d* and the glass coating thickness ([*D* − *d*]/2) are different.

The hysteresis loops of both samples were measured using the fluxmetric method at 114 Hz. As previously reported [43], such a method allows the recording of the hysteresis loop of magnetic microwires with high resolution. In this method, the samples were placed inside the pick-up coil and magnetized by 120 mm long and thin (8 mm in diameter) solenoid. This solenoid produced a homogeneous axial magnetic field *H*. For better comparison of the samples with different diameters and chemical compositions, the hysteresis loops were represented in terms of the normalized magnetization *M*/*M_o_* (*M_o_* being the magnetic moment of the sample at maximum magnetic field amplitude *H_o_*), dependent on magnetic field *H*.

The DW propagation (dependence of DW velocity on applied magnetic field H) was studied using a modified Sixtus–Tonks method [40]. To briefly describe it, the DW velocity *v* is evaluated from the electromotive force *EMF* signals, induced by the propagating single DW in three pick-up coils surrounding the microwire, separated by the same distance, *l*, and placed coaxially inside the magnetizing coil. In this case, the *v* value can be estimated from the time difference ∆*t*, at which the *EMF* signals are detected in each pick-up coil as [40,44]:

*v* = *l*/∆t (2)

The impedance *Z*, and its dependence on magnetic field *H*, were measured from the reflection coefficient *S*_11_, evaluated by the vector network analyzer (VNA) N5230A, as described elsewhere [32,33,34,35]. Use of a micro-strip sample holder allows measurement of the *Z* (*H*) dependence in a frequency range up to 1 GHz [45]. From *Z* values obtained for different *H* values, we evaluated the magnetic field dependences of the GMI ratio ∆*Z*/*Z*, as defined from Equation (1).

The frequency dependence of the maximum GMI ratio ∆*Z*/*Z_max_*, defined as the maximum ∆*Z*/*Z* value obtained for each given measurement frequency *f*, was also evaluated.

We evaluated as-prepared samples as well as the samples annealed in the furnace at annealing temperatures (*T_ann_*) 300, 400 and 500 °C for annealing time (*t_ann_*) 1 and 3 h. Additionally, we studied the samples subjected to Joule heating, flowing the DC current with the current values and *t_ann_* previously employed for such microwires [46].

The conditions of Joule heating were selected considering the current density *j*. In our previous publications, it was shown that Joule heating at *j* of 20 A/mm^2^ gives good results. Accordingly, we chose the current value *I* to be 15 and 40 mA for “thin” and “thick” samples, respectively.

## 3. Results and Discussion

- A substantial GMI ratio improvement by annealing;
- A remarkable effect of annealing on domain wall dynamics.

### 3.1. Hysteresis Loops

**Figure 1:**
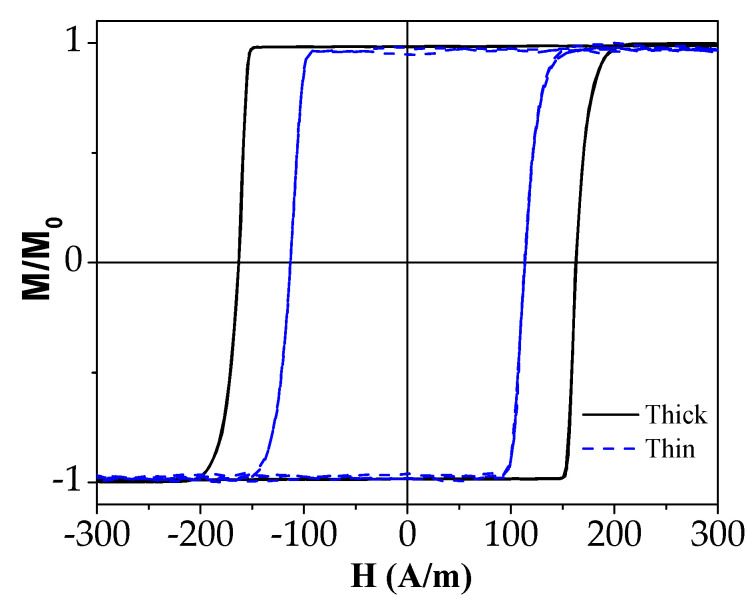
Hysteresis loops of as-prepared microwires.

The hysteresis loops of both studied as-prepared samples are provided in Figure 1. As expected for Fe-rich microwires with positive magnetostriction *λ_s_*, both as-prepared samples presented perfectly rectangular hysteresis loops.

The only unexpected observation was that the thinner microwires presented lower coercivity H_C_ (see Figure 1). There are several possible reasons for such lower coercivity with thinner diameter, such as a lower quenching rate caused by the thicker insulating glass coating. As previously shown [47], the glass coating does not absorb heat, hindering the heat exchange due to its low thermal conductivity.

**Figure 2:**
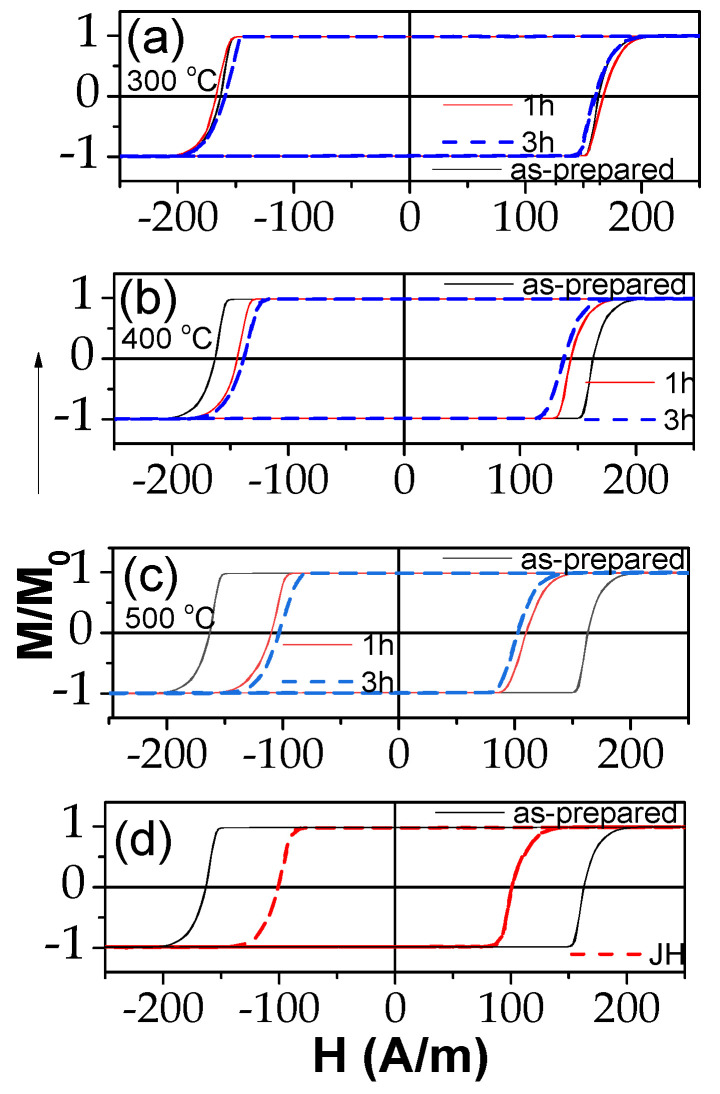
Effect of annealing at 300 (**a**), 400 (**b**) and 500 °C (**c**) and Joule heating (**d**) on hysteresis loops of Fe_72_B_13_Si_11_Nb_3_Ni_1_ microwires.

Conventional furnace annealing at 300–500 °C affected, if only slightly, the hysteresis loops of “thick” microwires (see Figure 2a–c). The hysteresis loop of samples annealed at 300 °C (1 h and 3 h) showed almost no change from their as-cast state (Figure 2a). However, higher annealing temperatures did result in bigger changes of the loops (Figure 2b,c). On the other hand, Joule heating (at 40 mA for 20 min) might be a more effective route for magnetic softening of “thick” microwires, as it achieved the lowest coercivity value observed (Figure 2d), among those obtained in annealing at 500 °C for one hour, in less than half the time.

**Figure 3:**
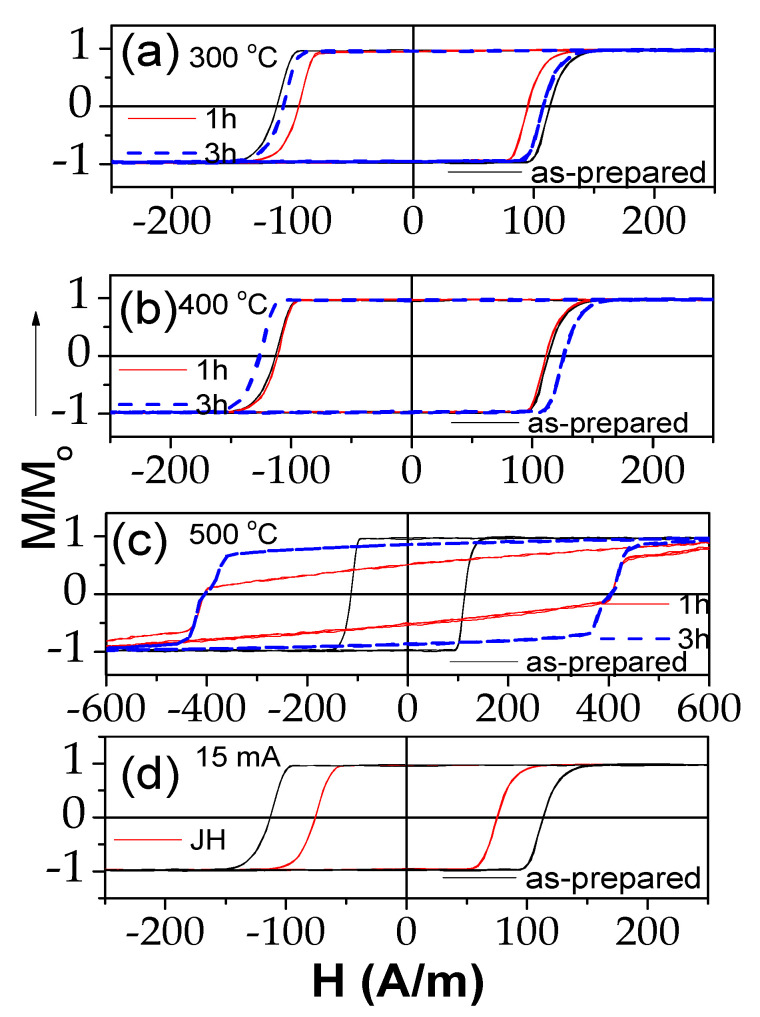
Effect of furnace annealing at 300 (**a**), 400 (**b**) and 500 °C (**c**) and Joule heating (**d**) on hysteresis loops of Fe_75_B_9_Si_12_C_4_ microwire.

Similarly, in “thin” Fe_75_B_9_Si_12_C_4_ microwire, Joule heating is the most effective for magnetic softening; the lowest coercivity was observed in Joule-heated samples (with 15 mA for 3 min) (Figure 3d). Some magnetic softening was also observed after annealing at 300 °C for one hour (Figure 3a). However, further increasing the annealing temperature and/or time resulted in magnetic hardening, with H_C_ values growing bigger with annealing (Figure 3a–c). The severe magnetic hardening observed for the sample annealed at 500 °C must be attributed to the beginning of crystallization of the alloy (Figure 3c). Crystallization of Fe_75_B_9_Si_12_C_4_ microwires has already been studied in several publications [35,36]. While heating at 10 K/min, the onset of crystallization was observed at about 522 K, whereas for *t_ann_* = 3.5 h of annealing, the crystallization was observed at *T_ann_* = 430 °C. In all cases except T_ann_ = 500 °C, the hysteresis loops of all annealed and Joule-heated samples maintained perfectly rectangular shapes.

**Figure 4:**
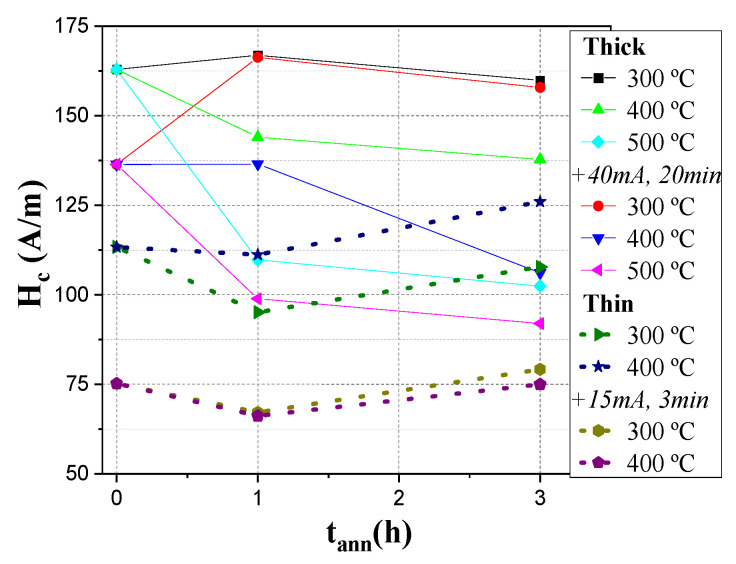
H_C_ dependence on annealing time. The “+” indicates the application of current annealing after conventional annealing.

A summary of the observed tendencies in the modification of the *H_C_* values after various thermal treatments is provided in Figure 4.

As can be concluded from the experimental results provided above, hysteresis loops of both studied samples were slightly affected by annealing at moderate *T_ann_* as well as by Joule heating. However, from previous publications it is known that annealing can substantially affect both DW dynamics and GMI effect performance [33,35,36].

Therefore, below we provide our experimental results on the effect of annealing on DW dynamics and GMI effect in both studied samples.

### 3.2. Domain Wall Dynamics

**Figure 5:**
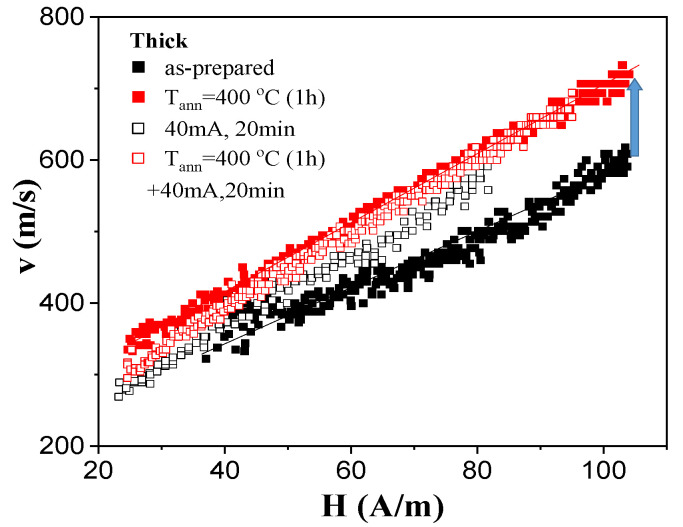
Effect of annealing on single DW dynamics of Fe_72_B_13_Si_11_Nb_3_Ni_1_ microwires.

**Figure 6:**
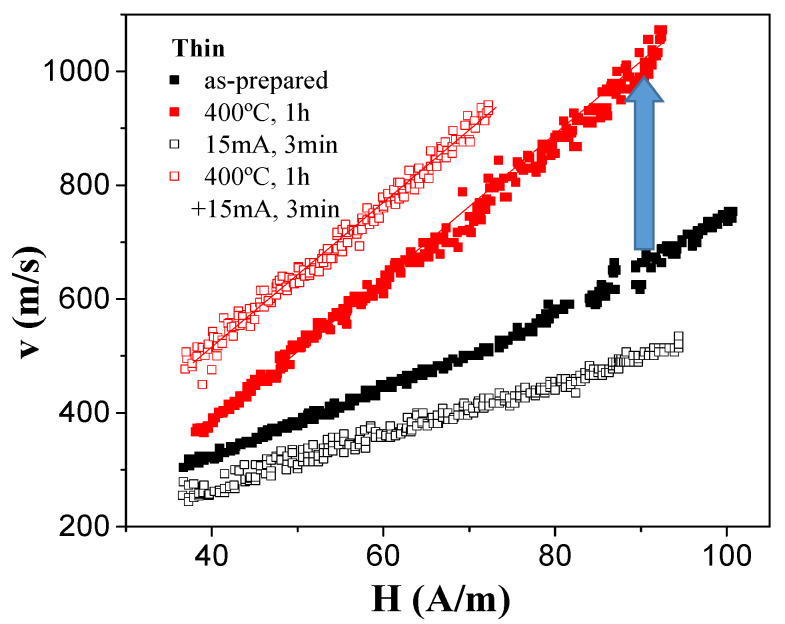
Effect of annealing on single DW dynamics of Fe_75_B_9_Si_12_C_4_ microwire.

The most relevant experimental results of annealing on DW dynamics for both studied samples are provided in Figure 5 and Figure 6 for “thick” and “thin” microwires, respectively. In all cases, the dependence of DW velocity *v* on magnetic field H can be roughly represented as a linear dependence:

*v* = *S*(*H* − *H*_0_) (3)

where *S* is the DW mobility, and *H*_0_ is the critical propagation field.

As can be appreciated from Figure 5, an increase in *v* and *S* values was observed for the “thick” Fe_72_B_13_Si_11_Nb_3_Ni_1_ microwire after thermal treatment. Although the increase in maximum *v* values from 500 to 700 m/s can be appreciated from Figure 5 (for the sample annealed at 400 °C for 1 h), *S* values increased only slightly from 4.3 to 5 m^2^/As.

More substantial changes in DW dynamics after annealing were observed for “thin” Fe_75_B_9_Si_12_C_4_ microwire. In this sample, an increase in maximum *v* values from 750 m/s to 1050 m/s was observed, while the *S* values increased from 7 to 12 m^2^/As upon conventional annealing (see Figure 6). Unexpectedly, a decrease in both *v* and *S* values was observed after Joule heating. However, Joule heating after conventional annealing allowed the increasing of *v* values at fixed *H* values (i.e., from 760 m/s to 900 m/s at H = 70 A/m), while the *S* value remained about 13 m^2^/As (see Figure 6).

Previously, the improvement of the DW dynamics, and, particularly, the DW mobility, was discussed elsewhere in terms of the relationship of the DW mobility and magnetoelastic anisotropy *K_me_* through the viscous damping coefficient *β*, given as [40,48,49]:

*S* = 2*μ*_0_*M_S_*/*β* (4)

where *μ*_0_ is the magnetic permeability of the vacuum and *M_S_* the saturation magnetization.

Generally, there are several contributions to viscous damping coefficient *β*, such as micro-eddy current contribution *βe* and the magnetic relaxation damping *βr*. The latter is commonly considered as the main factor affecting the DW dynamics in amorphous microwires and is related to the *K_me_* through the expression given as [48,49]:

*β_r_* ≈ 2*M_s_π*^−1^ (*K_me_*/*A*)^1/2^ (5)

where *A* is the exchange stiffness constant.

Consequently, the effect of thermal treatment on DW dynamics must be attributed to the relationship of *βr* and *K_me_*, given as:

*K_me_* = 3/2 *λ_s_σ*, (6)

where *σ*_=_ *σ_i_*_+_ *σ_a_*, and *σ_i_* and *σ_a_* are the internal and applied stresses, respectively.

### 3.3. Giant Magnetoimpedance (GMI) Effect

**Figure 7:**
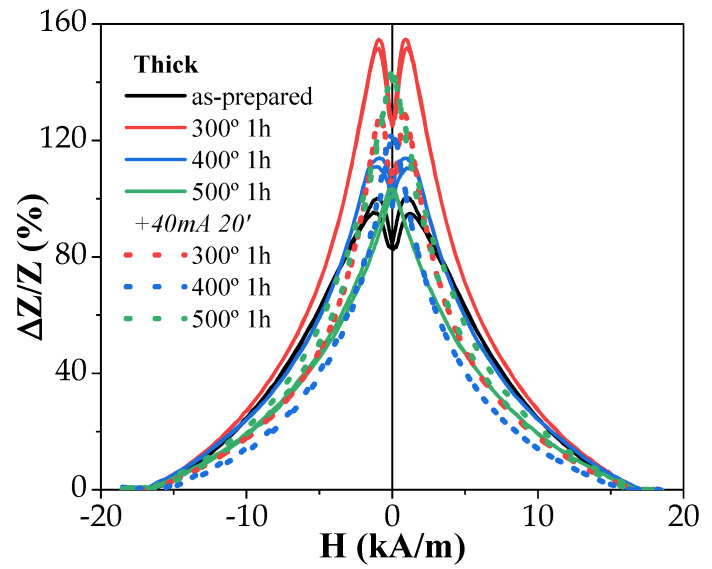
Effect of annealing on GMI effect performance of Fe_72_B_13_Si_11_Nb_3_Ni_1_ microwire measured at f = 200 MHz.

**Figure 8:**
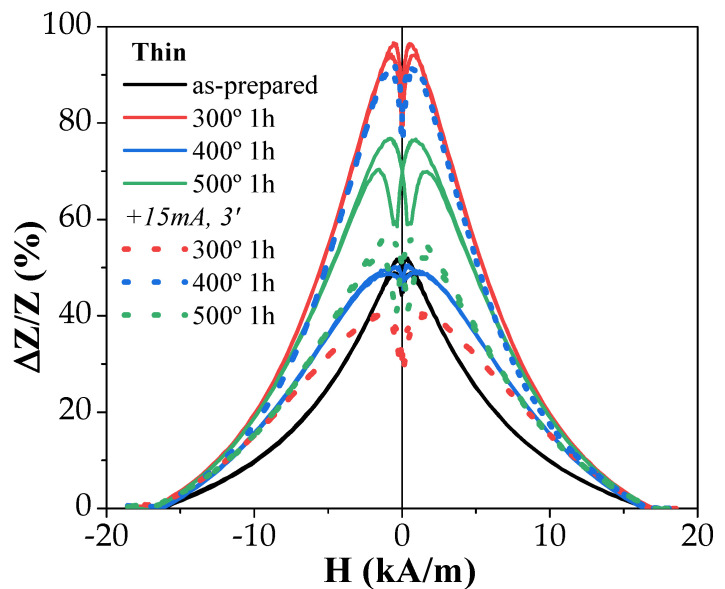
Effect of annealing on GMI effect performance of Fe_75_B_9_Si_12_C_4_ microwire measured at f = 200 MHz.

The experimental results on the influence of annealing on the GMI effect of both studied samples are provided in Figure 7 and Figure 8.

There were several features of the GMI effect, including:

(i) The GMI ratio of “thick” samples was rather large even in as-prepared samples;
(ii) In both studied samples, for almost all annealing conditions, an increase in ∆*Z*/*Z* values was observed after the process;
(iii) Both microwires exhibited a double-peak ∆*Z*/*Z*(*H*) dependence at high enough frequencies. Usually, these double-peak ∆*Z*/*Z*(*H*) dependencies are reported for Co-rich microwires [43,45], while single-peak ∆*Z*/*Z*(*H*) dependencies with ∆*Z*/*Z* maximum at *H* = 0 are predicted for microwires with axial magnetic anisotropy, such as ours (which is confirmed by the hysteresis loops’ character) [50]. Such single-peak ∆*Z*/*Z*(*H*) dependence was observed in “thick” samples annealed at high enough temperatures (see Figure 7).

The most probable cause for the double-peak ∆*Z*/*Z*(*H*) is the presence of a transverse magnetic anisotropy domain in the outer area of the metallic core of the studied microwires [43,43].

As-prepared “thick” samples showed a relatively high ∆*Z/Z_max_* ratio (~100%), higher than that of “thin” ones (~50%) (Figure 7 and Figure 8). However, in both microwires, annealing allowed an enhancement of the ∆*Z*/*Z*_max_ ratio from 50% to 100% in “thin” microwires and from 100% to a bit less than 160% for “thick” ones (Figure 7 and Figure 8).

**Figure 9:**
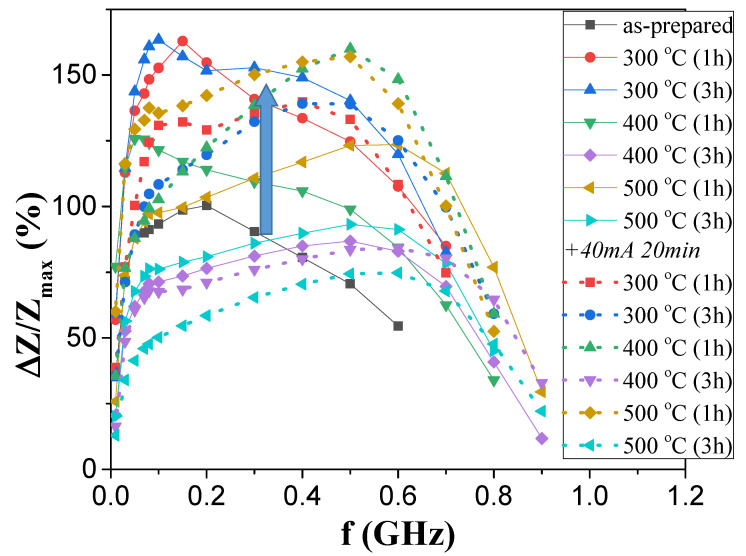
Effect of annealing on Δ*Z*/*Z_max_* (*f*) dependence of Fe_72_B_13_Si_11_Nb_3_Ni_1_ microwire.

**Figure 10:**
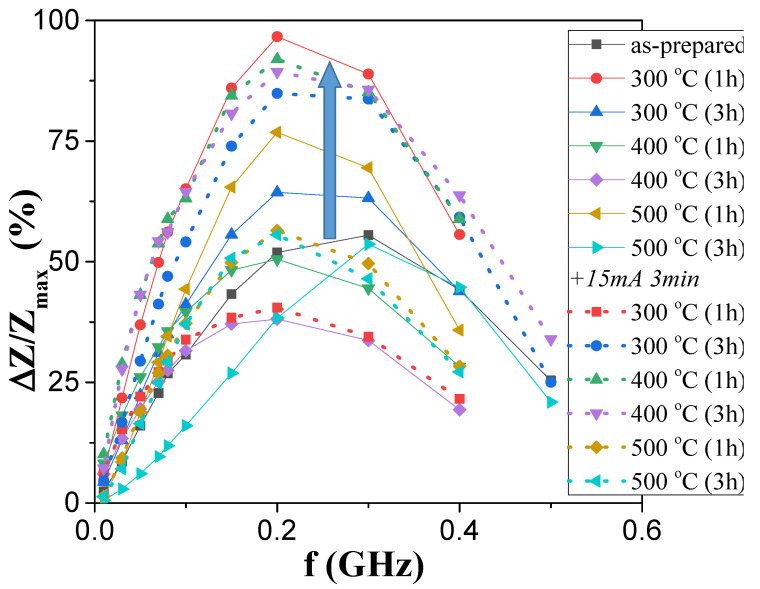
Effect of annealing on Δ*Z/Z_max_* (*f*) dependence of Fe_75_B_9_Si_12_C_4_ microwire.

Frequency dependencies of the ∆*Z*/*Z_max_* are provided in Figure 9 and Figure 10 for better representation of how annealing affected the ∆*Z*/*Z_max_* of “thick” and “thin” microwires, respectively.

When comparing ∆*Z*/*Z_max_* (*f*) dependencies of both studied samples, it became obvious that thicker microwires yield higher ∆*Z*/*Z_max_* values in the whole frequency range, as the highest ∆*Z*/*Z_max_* ratios achieved in annealed “thin” samples did not surpass those of the “thick” as-prepared sample.

However, it is relevant that the annealing provided an enhancement in the ∆*Z*/*Z_max_* ratios of both microwires. A more detailed analysis revealed some more complex behavior. As such, conventional annealing at 300 °C of the Fe_72_B_13_Si_11_Nb_3_Ni_1_ microwire resulted in the highest ∆*Z*/*Z_max_* values of the sample for both 1 and 3 h of processing, while annealing at 400 °C started to lower said values to levels similar to that of the as-prepared sample. The addition of current annealing after these processes yielded a negative effect in most cases as the GMI ratios values became lower, with the exception of the annealing with 400 and 500 °C for 1 h each, which resulted in an enhancement comparable to the one obtained at 300 °C.

The results shown in Figure 10 slightly differ from the above. While annealing at 300 °C for 1 h did indeed enhance the GMI ratios, further conventional annealing resulted in lower values, even below those obtained with as-prepared samples. However, these ratios could be brought back up to higher values as application of Joule heating on samples annealed for 3 h at 300 °C, and for 1 and 3 h at 400 °C, resulted in values comparable to the highest ones obtained for these processes.

As discussed elsewhere, the peculiarity of Joule heating is the presence of an Oersted magnetic field produced by the passing of an electrical current through the microwire [34,51,52]. Therefore, during the Joule heating, the sample is annealed in the presence of the circumferential magnetic field *H_circ_* (associated to the DC current I flowing through the microwire), also known as the Oersted field, given as [20]:

*H_circ_* = *Ir*/2*πR*^2^ (7)

where *I* is the current value, *r*—radial distance and *R*—microwire metallic nucleus radius.

Accordingly, *H_circ_* varies from zero on the microwire axis to the maximum value on the surface.

In the present case, the *I* values were selected to avoid the crystallization of the studied microwires. From previous studies, it is known that the current density is the main parameter that affects the sample temperature during the Joule heating [53,54]. Thus, Joule heating at *j* = 30–45 A/mm^2^ can produce the heating of amorphous materials up to 400 °C [53,54]. Therefore, we chose *j* ≈ 20 A/mm^2^. For the studied microwires (with *r* ≈ 7.6 and 23.95 µm), Equation (7) gives *H_circ_* ≈ 315 and 265 A/m on the surface of the metallic nucleus for *I* = 15 mA and 40 mA, respectively. These *H_circ_* values were superior to the *H_c_* values (see Figure 1). Therefore, during Joule heating, the surface layer of the magnetic microwire was magnetized by the circumferential magnetic field *H_circ_*. From previous studies, it is known that substantial magnetic anisotropy can be induced by magnetic annealing [55,56]. In the present case, Joule heating was associated with the annealing of the inner core, while the surface layer was subjected to annealing in a circumferential magnetic field. The GMI effect value and features are determined by the magnetic anisotropy in a surface layer. Therefore, despite the fact that the bulk hysteresis loops remained practically unchanged (see Figure 2 and Figure 3), in some cases, Joule heating could significantly affect the features and magnitude of the GMI effect [14]. Therefore, after Joule heating, a contribution of induced magnetic anisotropy associated with the Oersted field is expected.

Similarly, induced transverse magnetic anisotropy in the surface layer can be beneficial for the DW dynamics improvement in amorphous microwires [14].

On the other hand, annealing itself is the traditional way, allowing internal stresses relaxation. As discussed elsewhere, stresses relaxation associated with annealing can substantially affect the damping parameter [57]. The beneficial influence of internal stresses relaxation, as well as the change in the damping parameter, on DW dynamics is discussed elsewhere [58,59]. However, the GMI effect value also depends substantially on the value of the phenomenological damping parameter [50].

Additionally, annealing involves not only internal stresses relaxation, but also structural relaxation due to the atomic short-range order changes of amorphous materials [60,61,62]. The detailed description of various atomic mechanisms responsible for the changes in *T_c_* and other physical properties is provided elsewhere [60,62]. Such mechanisms include diffusion of structural defects, topological and compositional short-range atomic ordering and clustering [60,62]. Accordingly, various magnetic properties, such as magnetostriction or the Curie temperature of amorphous alloys, depend on the local atomic environment, the presence of clusters and even stresses [60,61,62]. Such variation of the magnetostriction coefficient *λ_s_*, or Curie temperature *T_c_*, upon annealing has been reported for amorphous ribbons [60,61,62] and has also recently been observed for amorphous microwires [63]. Obviously, such modification in *λ_s_* or *T_c_* upon annealing can substantially affect both the DW dynamics and GMI effect of studied microwires.

Accordingly, in certain cases, conventional furnace annealing and Joule heating can be useful for both GMI effect and DW dynamics optimization.

The overall conclusion is that the annealing of Fe-rich microwires under appropriate conditions is useful for the enhancement and attunement of GMI effect performance and DW dynamics optimization. Accordingly, appropriate postprocessing of Fe-rich microwires is beneficial for magnetic sensor and devices applications of Fe-rich microwires.

## 4. Conclusions

Routes for GMI effect and DW dynamics optimization were presented for two Fe-rich microwires with rather different metallic nucleus diameters prepared using the Taylor–Ulitovsky method. As-prepared thinner microwires possess better magnetic softness (lower coercivity values). However, annealing allows considerable improvement of both GMI effect and single DW dynamics.

Current annealing may prove to be a decent alternative to heat annealing as, with only some minutes of treatment, a more substantial magnetic softening can be achieved. The combination of both thermal treatments yields a further reduction in coercivity values, especially in thicker microwires.

Higher domain wall velocity values can be achieved in thinner Fe-rich microwires after appropriate annealing. However, even in thicker microwires, an improvement in DW dynamics upon annealing is observed.

Conventional annealing of both types of microwires at 300 °C for 1 h has been shown to yield the highest values of GMI ratios. Generally, the highest GMI ratios have been achieved in thicker microwires. While Joule heating used after conventional annealing is mostly detrimental, for specific cases, the GMI ratio values are once again enhanced after their lowering due to excessive annealing temperatures or times. This might prove to be an interesting option to “repair” the sensitivity of microwires used in magnetic sensing technologies at high temperatures.

Appropriate postprocessing of Fe-rich microwires is a relevant tool for making Fe-rich microwires suitable for magnetic sensor and devices applications.

## Figures and Tables

**Table 1:** Compositions and geometrical parameters of studied glass-coated microwires.

Sample	*d* (µm)	*D* (µm)	*ρ* = *d*/*D*	Chemical Composition
“Thick”	47.9	53.2	0.9	Fe_72_B_13_Si_11_Nb_3_Ni_1_
“Thin”	15.2	17.2	0.88	Fe_75_B_9_Si_12_C_4_

## Data Availability

Data available on request due to restrictions related to the developing projects.
